# Evaluation of gastrin-releasing peptide receptor, prostate-specific membrane antigen, and neurotensin receptor 1 as potential biomarkers for accurate prostate cancer stratified diagnosis

**DOI:** 10.1186/s13550-024-01116-3

**Published:** 2024-06-16

**Authors:** Ling Xiao, Zhihui Fang, Yongxiang Tang, Yanyan Sun, Zehua Zhu, Jian Li, Ming Zhou, Nengan Yang, Kai Zheng, Shuo Hu

**Affiliations:** 1 Department of Nuclear Medicine, Xiangya Hospital, Central South University, No.87 Xiangya Road, Changsha City, 410008 Hunan Province P.R. China; 2 Department of Nuclear Medicine, The Second Xiangya Hospital, Central South University, Changsha, 410008 China; 3 Department of Hematology, The Affiliated Cancer Hospital of Zhengzhou University and Henan Cancer Hospital, Zhengzhou, 450000 China; 4 National Clinical Research Center for Geriatric Disorders, Xiangya Hospital, Central South University, Changsha, 410008 China; 5 Key Laboratory of Biological Nanotechnology of National Health Commission, Xiangya Hospital, Central South University, Changsha, 410008 China

**Keywords:** Prostate cancer, Positron-Emission tomography, Prognosis, Diagnosis

## Abstract

**Background:**

Studies on single-target PET imaging of gastrin-releasing peptide receptor (GRPR), prostate-specific membrane antigen (PSMA), or neurotensin receptor 1(NTR1) have been reported. However, the performance of these three targets in the progression of PCa remains unclear. Our study aims to compare the expression of GRPR, PSMA, and NTR1 in patients with prostatic intraepithelial neoplasia (PIN), prostate cancer (PCa), and lymph node metastasis. We synthesized molecular probes targeting the markers to achieve a non-invasive precise detection of PCa patients with PET/CT imaging.

**Methods:**

In this study, the expression of GRPR, PSMA, and NTR1 was evaluated by immunohistochemistry in 34 PIN, 171 PCa, and 22 lymph node metastasis tissues of patients. The correlation between their expression and the clinicopathological parameters of PCa patients was assessed. Sixteen PCa patients with different Gleason scores (GS) underwent dual-tracer (^68^Ga-NOTA-RM26 and ^68^Ga-NOTA-PSMA617) PET/CT.

**Results:**

In the PIN stage, the expression of GRPR was significantly higher than that of PSMA and NTR1 (*P* < 0.001), while NTR1 expression was significantly higher than PSMA and GRPR expression in primary PCa (*P* = 0.001). High PSMA expression in PCa patients was associated with shorter progression-free survival (*P* = 0.037) and overall survival (*P* = 0.035). PCa patients with high GS had higher tumor uptake of ^68^Ga-NOTA-PSMA617 than those with low GS (*P* = 0.001), while PCa patients with low GS had higher tumor uptake of ^68^Ga-NOTA-RM26 than those with high GS (*P* = 0.001).

**Conclusions:**

This study presents three novel biomarkers (PSMA, GRPR, and NTR1) as imaging agents for PET/CT, and may offer a promising approach for non-invasive precise detection and Gleason grade prediction of PCa patients.

**Supplementary Information:**

The online version contains supplementary material available at 10.1186/s13550-024-01116-3.

## Introduction

Prostate cancer (PCa) is one of the most common tumors in adult men [1]. Precise prostate cancer detection and Gleason grade prediction have great significance for clinical treatment and decision making. PCa diagnosis is based on the gold standard procedure of transrectal ultrasound (TRUS) or magnetic resonance imaging (MRI) guided needle biopsy of the prostate. However, puncture biopsy is an invasive examination that may have risks such as bleeding and infection [2], On the other hand, The prostate biopsy Gleason grade frequently differs from the radical prostatectomy (RP) grade [3, 4]. MRI play a crucial role in the clinical diagnosis and treatment of PCa. While multiparametric magnetic resonance imaging (mpMRI) has emerged as the preferred imaging technique for diagnosing localized prostate cancer [5], it exhibits limitations in terms of diagnostic specificity (41%) and sensitivity for local PCa staging [6]. Additionally, mpMRI faces challenges in detecting small foci in the central gland of the prostate, leading to a misdiagnosis rate of 35% for clinically significant PCa [7]. Therefore, alternative imaging modalities are necessary to overcome the limitations of mpMRI in PCa diagnosis and staging. In recent years, positron emission tomography (PET) imaging of tumor-specific targets has provided a better option for detecting tumors, identifying biological characteristics, and evaluating curative effects.

Prostate-specific membrane antigen (PSMA), a transmembrane glycoprotein, is overexpressed on malignant prostate tumor cells. In recent years, PSMA has become a preferred target for positron emission tomography (PET) imaging and radionuclide therapy for PCa, with high sensitivity and specificity for PCa detection or localization [8, 9]. Several studies suggest that ^68^Ga-PSMA PET/CT may accurately discriminate clinically significant prostate cancer (csPCa) from benign prostate diseases, ^68^Ga-PSMA-11 PET/CT should be the favored choice for primary staging of PCa in patients with GS > 7 or PSA levels ≥ 10 ng/ml [10, 11], but its detection of prostate cancer lesions with Gleason score = 6 is limited.

Gastrin-releasing peptide receptor (GRPR) is a G protein-coupled receptor, which is highly expressed on the surface of PCa cells [12]. Overexpression of GRPR and GPRR signaling can stimulate growth of both androgen-dependent and -independent prostate cancer cells, in addition, it can indirectly promote angiogenesis and promote the mitosis of prostate cancer cells. Bufalin also participates in the expression of some metalloproteinases (such as MMP-9), which stimulate the remodeling of extracellular matrix, thereby increasing the tumor’s invasive potential [13, 14]. Studies have shown that GRPR expression in atypical glands gradually increases during the progression from normal prostate tissue to high-grade intraepithelial neoplasia (HG-PIN); GRPR expression has also been reported to be inversely correlated with PSMA expression [15]. These findings suggested that GRPR may be a key target to compensate for the deficiency of PSMA in GS = 6 prostate cancer.

It has been reported that neurotensin (NT) and neurotensin receptors (NTRs) could play key roles in prostate cancer progression, especially when deprived of androgens [16], and in the development of castration-resistant PCa with neuroendocrine differentiation (NED) [17]. Our previous study found 91.8% of PCa tissues with moderate to high expressions of NTR1, suggesting that NTR1 could be a promising biomarker in prostate cancer research.

Studies on single-target PET imaging of NTR1, GRPR, or PSMA have been reported [18, 19]. However, a single target may not fully reflect the occurrence and development of PCa. Additionally, the performance of these three targets in the progression of PCa remains unclear. Therefore, it is necessary to explore molecular markers representing different stages of PCa to better stratify prostate cancer and potentially guide more accurate clinical management decisions.

In this study, we hypothesize that the expression levels of NTR1, GRPR and PSMA may differ in the high-level prostatic intraepithelial neoplasia (PIN) stage of prostate disease, the primary focus of PCa and the metastatic stage of PCa. Our second hypothesis is that PET/CT imaging targeting NTR1, GRPR, and PSMA could achieve non-invasive stratification of PCa-bearing models and prostate cancer lesions in vivo in patients, providing experimental and preliminary clinical data for non-invasive stratified diagnosis of PCa patients in the future.

Thus, the main objective of this study was to evaluate alternative targets for the better identification of intraprostatic lesions. We synthesized molecular probes targeting the markers to achieve non-invasive precise detection and Gleason grade prediction of prostate cancer patients with PET/CT imaging.

## Methods

### Study design

Our strategy was based on a sequential approach: First, we retrospectively performed PSMA, GRPR and NTR1 IHC in PIN, PCa, and lymph node metastatic lesions of prostate cancer; collected clinical pathological data from patients to analyze the relationship between the expression of GRPR, PSMA, and NTR1 proteins and clinical pathology. Next, we screened human prostate cancer cell lines with high expression of GRPR, PSMA or NTR1 by Real-Time PCR and Western blot for establishing the xenograft tumor model. Then we synthesized three imaging drugs targeting GRPR, PSMA and NTR and performed animal PET/CT imaging, which laid the foundation for the application of three targeted imaging drugs in clinical patient imaging. Finally, in order to further verify the stratify value of PET imaging agents targeting PSMA and GRPR for different GS PCa patients, we conducted dual-target PET imaging on patients with different GS.

### Patients

A total of 227 specimens of prostate tissue from 219 patients were consecutively collected from the patients underwent transurethral prostate resection or radical resection of PCa from January 2012 to January 2020 in the Department of Pathology at the Second Xiangya Hospital of Central South University. The tissue samples of PIN are from patients after transurethral prostate resection, the PCa tissue samples are from patients after radical resection of PCa, and the tissue samples of lymph node metastasis are from patients after radical resection of PCa with lymph node dissection or lymphadenectomy (The primary lesion can be confirmed as prostate cancer by puncture). Inclusion criteria for this retrospective study were: (a) no patient had received hormonal treatment, chemotherapy, or radiotherapy prior to surgery; and (b) The clinicopathological data of prostate cancer patients is complete. The following patients were excluded: (a) those with other combined primary malignancies or a history of malignancies; and (b) those who were diagnosed by puncture. The endpoints of interest were overall survival (OS) and progression-free survival (PFS). Patient age, total prostate-specific antigen (tPSA), pathological tumor (pT) stage, pathological nodal (pN) stage, margin status, and GS were evaluated. Ethical approval for this retrospective study was received from the institutional review board of the Second Xiangya Hospital. Informed consent was obtained from all participants of all 227 specimens or legal guardians included in the study.

### Immunohistochemical staining

Immunohistochemical staining was performed to confirm GRPR, PSMA, and NTR1 expression in the PIN, Primary and metastatic lesions of human PCa. Paraffin sections of the samples were then incubated with anti- GRPR, PSMA, or NTR1 antibodies (ab39883, ab19071, ab117592, Abcam).

### Cells and animals

To achieve the visualization of PET/CT molecular imaging for prostate cancer, we further synthesized molecular probes targeting GRPR, PSMA, and NTR1 for parallel in vivo imaging in mice. First, we selected three human prostate cancer cell lines: PC-3, LNCaP, and DU145, the cell lines with high expression of GRPR, PSMA or NTR1 were screened by Real-Time PCR and Western blot, respectively, and the xenograft tumor model was established. Then we synthesized three imaging drugs targeting GRPR, PSMA and NTR and performed animal PET/CT imaging, which laid the foundation for the application of three targeted imaging drugs in clinical patient imaging.

Human PCa cell lines, DU145 (brain metastasis lesion of human prostatic adenocarcinoma), LNCaP (lymph node metastasis lesion of human prostatic adenocarcinoma), and PC-3 (bone metastasis lesion of human prostatic adenocarcinoma) [20], were obtained from the National Collection of Authenticated Cell Cultures of China. And were cultured in low-glucose Dulbecco’s Modified Eagle Medium (Gibco), Roswell Park Memorial Institute (RPMI) 1640 medium (Gibco), and Ham’s F-12 K (Kaighn’s) medium (Gibco), respectively, supplemented with 10% fetal bovine serum. Cells were maintained in a 5% CO_2_ humidified incubator at 37 °C.

RT-PCR and Western blotting analysis was used to evaluate the expression of GRPR, PSMA, and NTR1in PC-3, LNCaP, and DU145 cells. The primary antibodies were the same as those used in immunohistochemistry (ab39883, ab19071 and ab117592).

### Xenograft tumor model

For the mouse xenograft, LNCaP cells (5 × 10^6^ cells in 100 ul RPMI-1640 medium enriched) were subcutaneously injected into the shoulder of male nonobese diabetic/severe combined immunodeficiency (NOD-SCID) mice. PC-3 cells (5 × 10^6^ in 200 ul of Ham’s F-12 K [Kaighn’s] medium) were subcutaneously injected into the shoulder of male BALB/c nude mice, there were at least 4 mice in each group. All experiments were performed in accordance with the guidelines for experimental animal use of Central South University. The protocol was approved by the ethics committee of Central South University [NO:2018sydw0251].

### Radiolabeling and quality control

The precursor GRPR(NOTA-RM26) and NTR1(NOTA-NT) were obtained from GL Biochem Company (China), the precursor PSMA(NOTA-PSMA617) was obtained from Huayi Isotopes Company (China). GRPR, PSMA and NTR1 were radiolabeled with an automated module (ITM). The peak times of ^68^Ga-NOTA-RM26, ^68^Ga-NOTA-PSMA617, and ^68^Ga-NOTA-NT were 8.72, 7.32, and 10.89 min, respectively (Supplemental Fig. 1, Supplemental Fig. 2). The purity of the products was more than 99%.

### Animal imaging

Mice were anesthetized by isoflurane inhalation before injecting 3.7 MBq of ^68^Ga-NOTA-RM26, ^68^Ga-NOTA-PSMA617, or ^68^Ga-NOTA-NT. Small-animal PET/CT (Mediso, Budapest, Hungary) imaging was performed 30 min after the injection. After fixation, dehydration, paraffin embedding, and sectioning, the expression of GRPR, PSMA, and NTR1 was detected by immunohistochemical staining.

### PET/CT imaging in PCa patients

The human pilot study prospectively enrolled sixteen patients with newly diagnosed, biopsy-proven PCa from June 2019 to June 2021. ^68^Ga-NOTA-PSMA617 and ^68^Ga-NOTA-RM26 PET/CT were performed before prostatectomy. Individuals were excluded from the study based on the following exclusion criteria: (a) GS = 7 or outcome not available; and (b) recent prior initiation of systemic treatment such as androgen deprivation therapy (ADT), chemotherapy, or radiotherapy. Patients were categorized into 2 different groups: low GS (GS = 6) and high GS (GS ≥ 8), according to their GS. The study protocol was approved by the Ethics Committee of the Xiangya Hospital of Central South University. Sixteen participants signed the informed consent.

Each patient received intravenous injection of ^68^Ga-NOTA-PSMA617 (median dose, 166.32 MBq; range, 121.36–193.14 MBq) and ^68^Ga-NOTA-RM26 (median dose, 174.69 MBq; range, 118.77–196.84 MBq) in two different days (time interval < 3 days). The radiochemical purity of ^68^Ga-NOTA-RM26 and ^68^Ga-NOTA-PSMA617 were greater than 99%. The images were reconstructed and independently reviewed by two experienced nuclear medicine physicians with consensus on a GE AW 4.6 workstation (Waukesha). The volumes of interest (VOIs) were then drawn to quantify the maximum standardized uptake value (SUVmax).

### Statistical analysis

The qualitative data were tested using the chi-square test or Fisher’s exact test as appropriate, and the grade data between 2 independent samples were tested using the Mann-Whitney test. Analyses of the PFS and the OS were performed using Kaplan–Meier estimates and compared in a log-rank test. To assess risk factors, a Cox proportional hazard regression model was used. A *P* value < 0.05 was considered statistically significant. SPSS 22.0 (IBM) software was used for analysis.

## Results

### Immunohistochemical staining

**Fig. 1:**
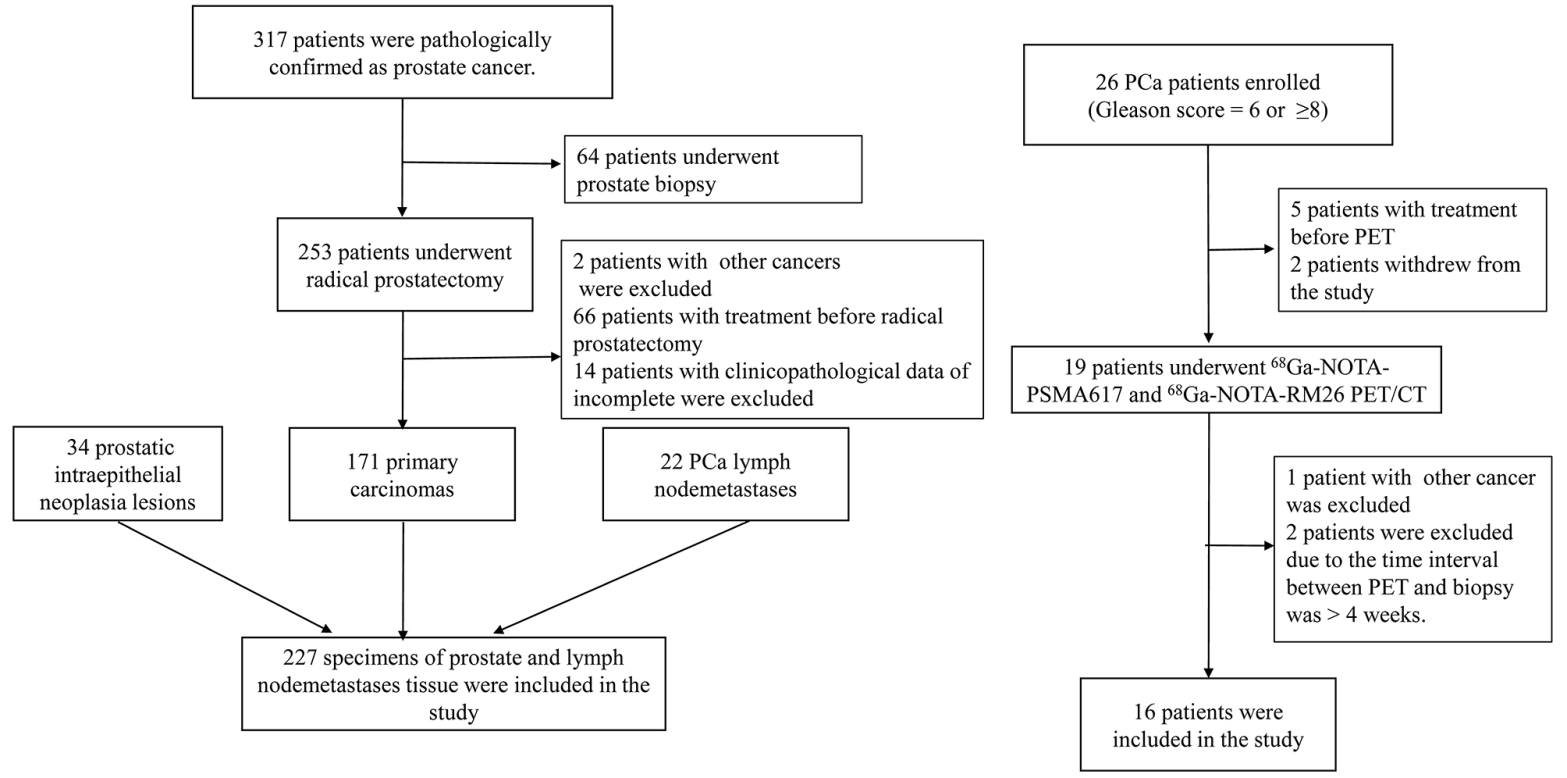
Study flowchart with excluded patients and reasons for exclusion

**Fig. 2:**
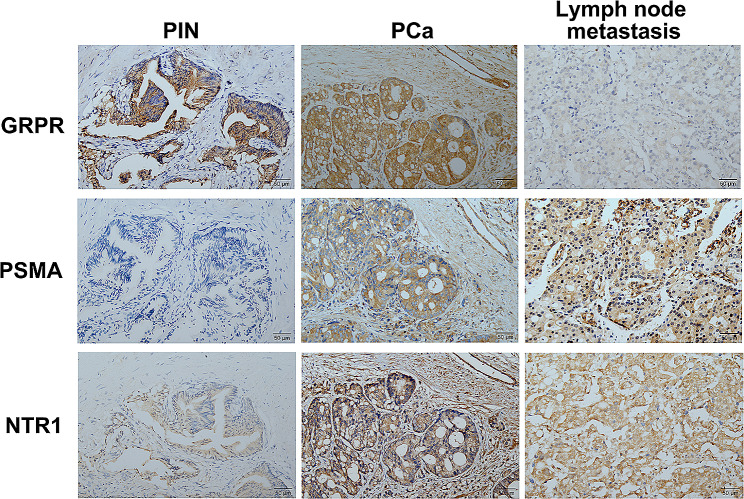
Representative immunohistochemistry staining of GRPR, PSMA, and NTR1 in PIN, PCa, and lymph node metastasis. 200×

The expression of GRPR, PSMA, and NTR1 was evaluated in tissue sections of 34 specimens of PIN, 171 specimens of PCa, and 22 specimens of lymph node metastasis by performing immunohistochemistry staining (Fig. 1). The median age of the patients was 66 years (range 46–95 years), and the median follow-up time was 41 months (range 15–92 months). In 34 PIN specimens, strong and moderate GRPR-, PSMA-, and NTR1-positive expression was observed (79.4%, 32.4%, and 2.9%, respectively) (Table 1; Fig. 2), and the expression of GRPR was significantly higher than those of NTR1 and PSMA (*P* < 0.05). Similarly, NTR1 expression was higher than another two in the 171 PCa specimens (*P* < 0.01), but there was no significant difference between GRPR and PSMA (*P* = 0.322). In tissue sections of 22 lymph node metastasis specimens, the expression of NTR1 and PSMA was significantly higher than that of GRPR (*P* < 0.01), with no significant difference between NTR1 and PSMA (*P* = 0.483). Out of 171 prostate cancer patients, two patients with neuroendocrine differentiation prostate cancer (GS = 5 + 4 and GS = 5 + 3) did not express PSMA (see Table 2), one of whom was positive for CgA, CD56, Syn, and NSE, while the other was positive for CgA and CD56, and both of them exhibited positive NTR1 and GRPR, with especially high NTR1 expression.

**Table 1:** Expressions of PSMA, GRPR, and NTR1 in PIN, PCa, and lymph node metastasis detected by immunohistochemistry

	PIN (*N* = 34)		*P*-Value	PCa(*N* = 171)		*P*-Value	lymph node metastasis (*N* = 22)		*P*-Value
	Low- Expression N (%)	High- Expression N (%)		Low- Expression N (%)	High- Expression N (%)		Low expression N (%)	High- Expression N (%)	
GRPR	7 (20.6)	27 (79.4)	< 0.001	40 (23.4)	131 (76.6)	0.001	11 (73.3)	4 (26.7)	< 0.001
PSMA	33 (97.1)	1 (2.9)		48 (28.1)	123 (71.9)		2 (11.8)	15 (88.2)	
NTR1	23 (67.6)	11 (32.4)		21 (12.3)	150 (87.7)		5 (22.7)	17 (77.3)	

**Table 2:** Expression of PSMA, GRPR, and NTR1 in 2 cases of PCa with neuroendocrine differentiation

NO.	GS	Ki-67	tPSA(ng/mL)	PSMA	GRPR	NTR1
1	5 + 4	60%	61.38	Negative	Strong positive	Strong positive
2	5 + 3	10%	10.32	Negative	Weak positive	positive

**Fig. 3:**
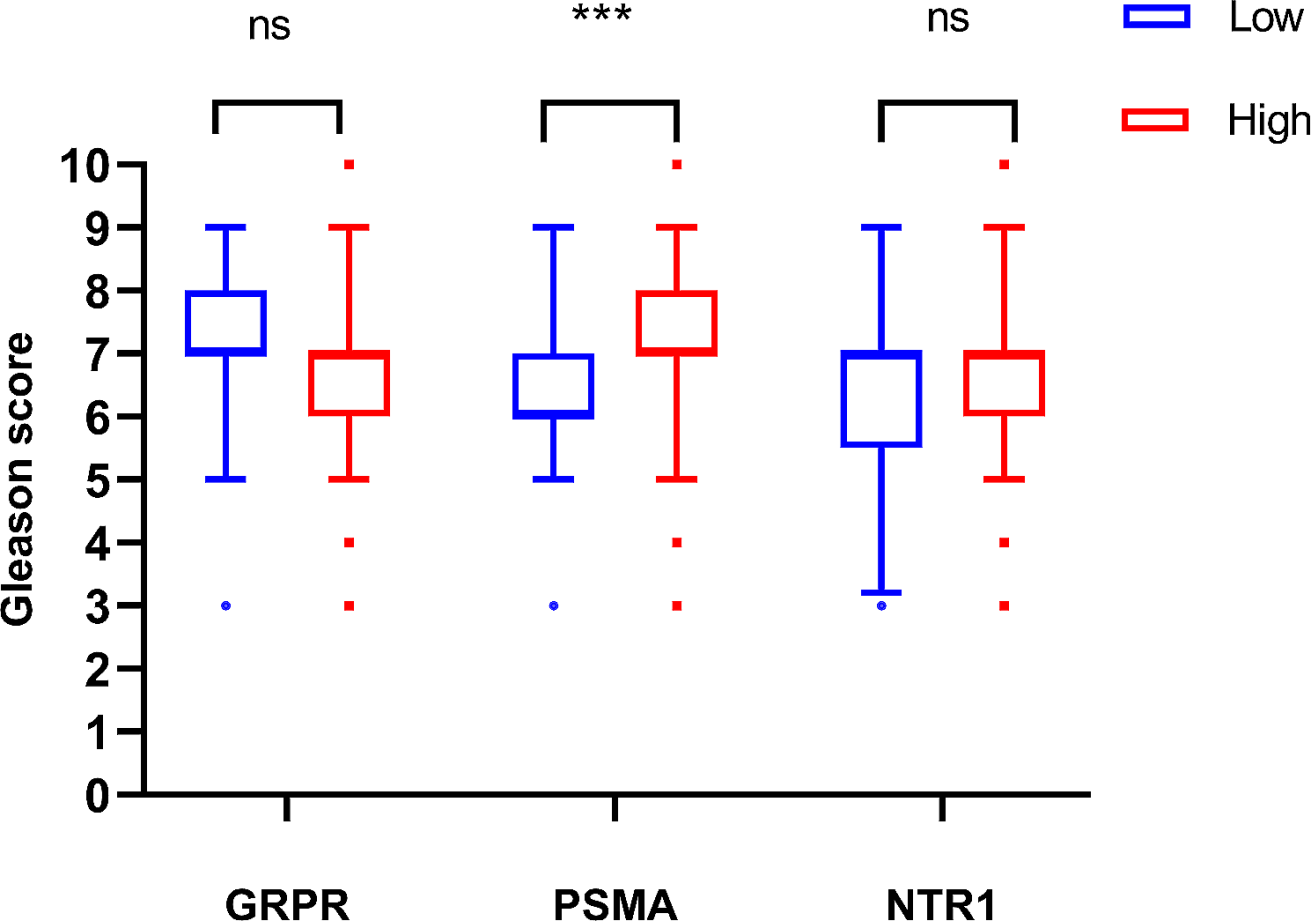
Comparison of Gleason score in primary tumors of diferent GRPR, PSMA, and NTR1 expression subgroups

### Correlation between clinicopathological characteristics and PSMA, GRPR, and NTR1 expressions

The correlations between GRPR, PSMA, and NTR1 expressions and clinicopathological characteristics in 171 patients with PCa are summarized in Table 3. PSMA expression had a positive correlation with GS (*P* < 0.001), but NTR1 expression was not correlated with any clinicopathological parameters. In 84 patients subjected to laparoscopic surgery, the margin status is described in pathological results. The positive margin of the GRPR high-expression group was significantly higher than the GRPR low-expression group (*P* = 0.038). The comparison of GS of all subgroups is illustrated in Fig. 3, the GS of patients with high PSMA expression was significantly higher than that of patients with low PSMA expression (*P* < 0.001).

**Fig. 4:**
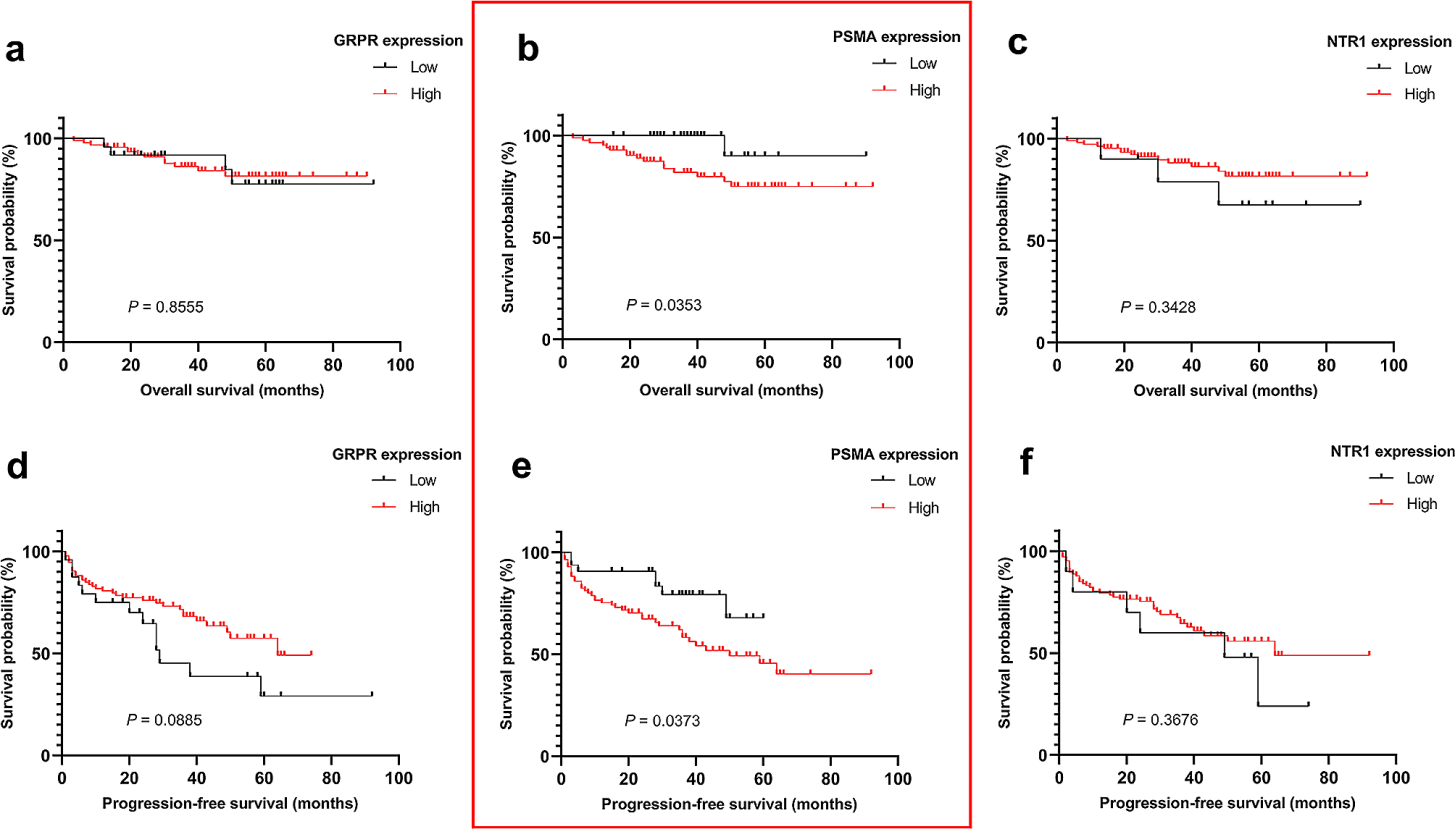
The relationship between GRPR, PSMA, and NTR1 expression and survival in patients with prostate cancer. Kaplan–Meier analysis of the difference in OS (a. GRPR, b. PSMA and c. NTR1) and PFS between high and low expression of (d. GRPR, e. PSMA and f. NTR1). Patients with low PSMA-expressing lesions have a better survival

**Table 3:** Correlation between GRPR, PSMA, and NTR1 expressions on IHC with clinicopathological features of patients with PCa

Variable	*N*	GRPR		*P*-Value	PSMA		*P*-Value	NTR1		*P*-Value
		Low- Expression *N* (%)	High- Expression *N* (%)		Low- Expression *N* (%)	High- Expression *N* (%)		Low- Expression *N* (%)	High- Expression *N* (%)	
**Age**				0.366			0.868			0.166
< 65years	66	13 (19.7)	53 (80.3)		19 (28.8)	47 (71.2)		11 (16.7)	55 (83.3)	
≥ 65years	105	27 (25.7)	78 (74.3)		29 (27.6)	76 (72.4)		10 (9.5)	95 (90.5)	
**tPSA**				0.835			0.854			0.597
≤ 10ng/mL	66	16 (24.2)	50 (75.8)		18 (27.3)	48 (72.7)		7 (10.6)	59 (89.4)	
> 10ng/mL	105	24 (22.9)	81 (77.1)		30 (28.6)	75 (71.4)		14 (13.3)	91 (86.7)	
**GS**				0.207			< 0.001			0.156
≤ 6	52	9 (17.3)	43 (82.7)		25 (48.1)	27 (51.9)		9 (17.3)	43 (82.7)	
7	80	20 (25.0)	60 (75.0)		18 (22.5)	62 (77.5)		9 (11.3)	71 (88.8)	
≥ 8	39	11 (28.2)	28 (71.8)		5 (12.8)	34 (87.2)		3 (7.7)	36 (92.3)	
**pT Stage**				0.448			0.230			0.355
≤T2	98	25 (25.5)	73 (74.5)		31 (31.6)	67 (68.4)		14 (14.3)	84 (85.7)	
≥T3	73	15 (20.5)	58 (79.5)		17 (23.3)	56 (76.7)		7 (9.6)	66 (90.4)	
**pN Stage**				0.957			0.271			1.000
N0	160	38 (23.8)	122 (76.3)		47 (29.4)	113 (71.6)		20 (12.5)	140 (87.5)	
N1	11	2 (18.2)	9 (81.8)		1 (9.1)	10 (90.9)		1 (8.3)	10 (91.7)	
**Margin status**				0.038			0.856			0.594
Negative	64	20 (31.3)	44 (68.8)		21 (32.8)	43 (67.2)		8 (12.5)	56 (87.5)	
Positive	20	1 (5.0)	19 (95.0)		7 (35.0)	13 (65.0)		1 (5.0)	19 (95.0)	

### Survival analysis

The expressions of GRPR, PSMA, and NTR1 were examined in the context of survival in 117 patients (Fig. 4). There was no significant difference in the PFS and OS between the low- and high-expression groups of GRPR and NTR1, but there was a significant difference in the PFS and OS between the low- and high-expression groups of PSMA (log-rank test: *P* = 0.037 and *P* = 0.035). The median PFS in the PSMA high- and low-expression groups was 50 ± 10.65 and not reached, while the median OS of the PSMA high- and low-expression groups was not reached, the average OS of the PSMA high- and low-expression groups was 75.76 ± 3.59 and 85.8 ± 3.98, respectively. Multivariate analysis by Cox proportional hazards model revealed that the GS constituted one of independent prognostic factors for PFS, with hazard ratio of 4.15 (95% confidence interval, 1.27–13.71, *P* = 0.018).

**Fig. 5:**
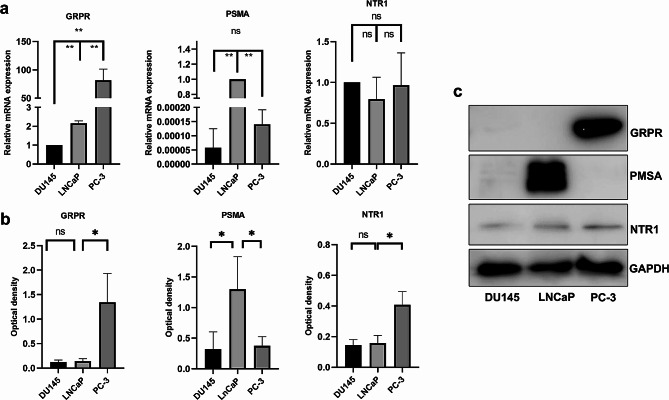
Expression of PSMA, GRPR, and NTR1 in PCa cell lines. RT-PCR (a) and Western blotting (b, c) analysis of GRPR, PSMA, and NTR1 expression in DU145, LNCaP, and PC-3 cell lines. *,P < 0.005, **,P<0.001. b. Quantitative analysis of Western blot bands.

### GRPR, PSMA, and NTR1 expression in PCa cell lines

We used quantitative RT-PCR and Western blot to measure mRNA and protein expressions of GRPR, PSMA, and NTR1 in LNCaP, PC-3 and DU145 cell lines. As displayed in Fig. 5-a, RT-PCR results indicated high expressions of GRPR mRNA and protein in PC-3 cells and high expression of PSMA mRNA and protein in LNCaP cells. the NTR1 protein level was significantly higher in PC-3 cells than that in another 2 cell lines (*P* < 0.05) (Fig. 5-b and -c, Supplemental Fig. 3).

### PET/CT imaging on small animals

**Fig. 6:**
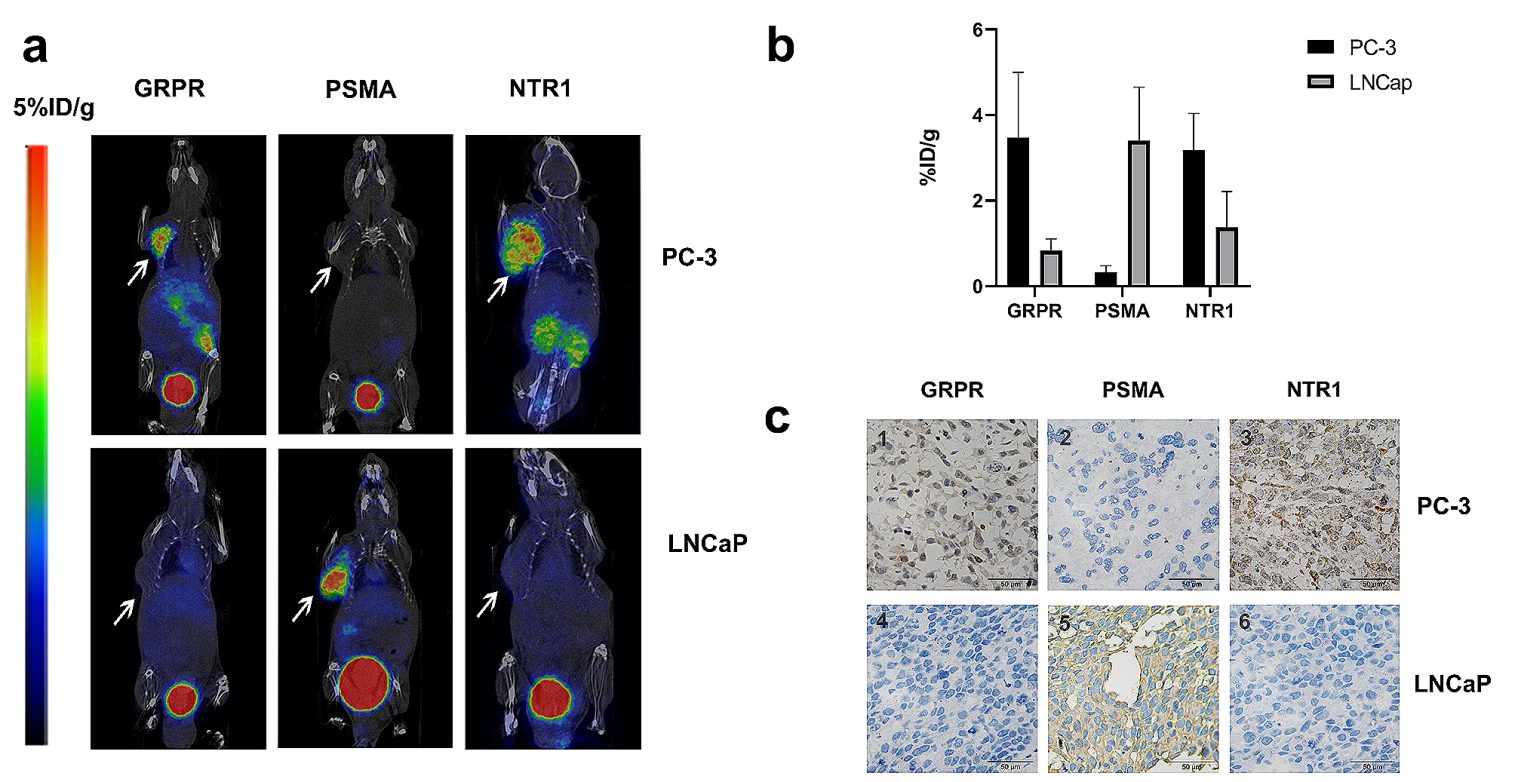
Micro PET/CT imaging of mice and immunohistochemistry. Representative PET/CT images (**a**) and quantitative analysis (**b**) of ^68^Ga-NOTA-RM26, ^68^Ga-NOTA-PSMA617, and ^68^Ga-NOTA-NT in PC-3 and LNCaP tumor models at 30 min post-injection. The white arrow indicates the tumor site. **c**. Representative immunohistochemistry staining of GRPR, PSMA, and NTR1 in PC-3 and LNCaP tumor tissues. 400**×**

The expression of GRPR, NTR1, and PSMA in PC-3 and LNCaP mouse xenografts was evaluated using ^68^Ga-labeled NOTA-RM26, NOTA-PSMA617, and NOTA-NT. As shown in Fig. 6-a, b, tumor uptake was observed 30 min post-injection. The high uptake and low background of ^68^Ga-NOTA-RM26 and ^68^Ga-NOTA-NT in the PC-3 tumor model (3.47 ± 1.532%ID/g, 3.18 ± 0.855%ID/g) and of ^68^Ga-NOTA-PSMA617 in the LNCaP tumor model (3.4 ± 1.252%ID/g) were observed. The expression of GRPR, PSMA, and NTR1 in PC-3 and LNCaP tumor tissues was examined by Immunohistochemical staining, and the results were consistent with PET/CT imaging (Fig. 6-c). GRPR and NTR1 were highly expressed in PC-3 transplanted tumors, and LNCaP transplanted tumors exhibited high expression of PSMA, but there was no significant expression of PSMA in PC-3 transplanted tumors.

### PET/CT imaging of PCa patients

**Fig. 7:**
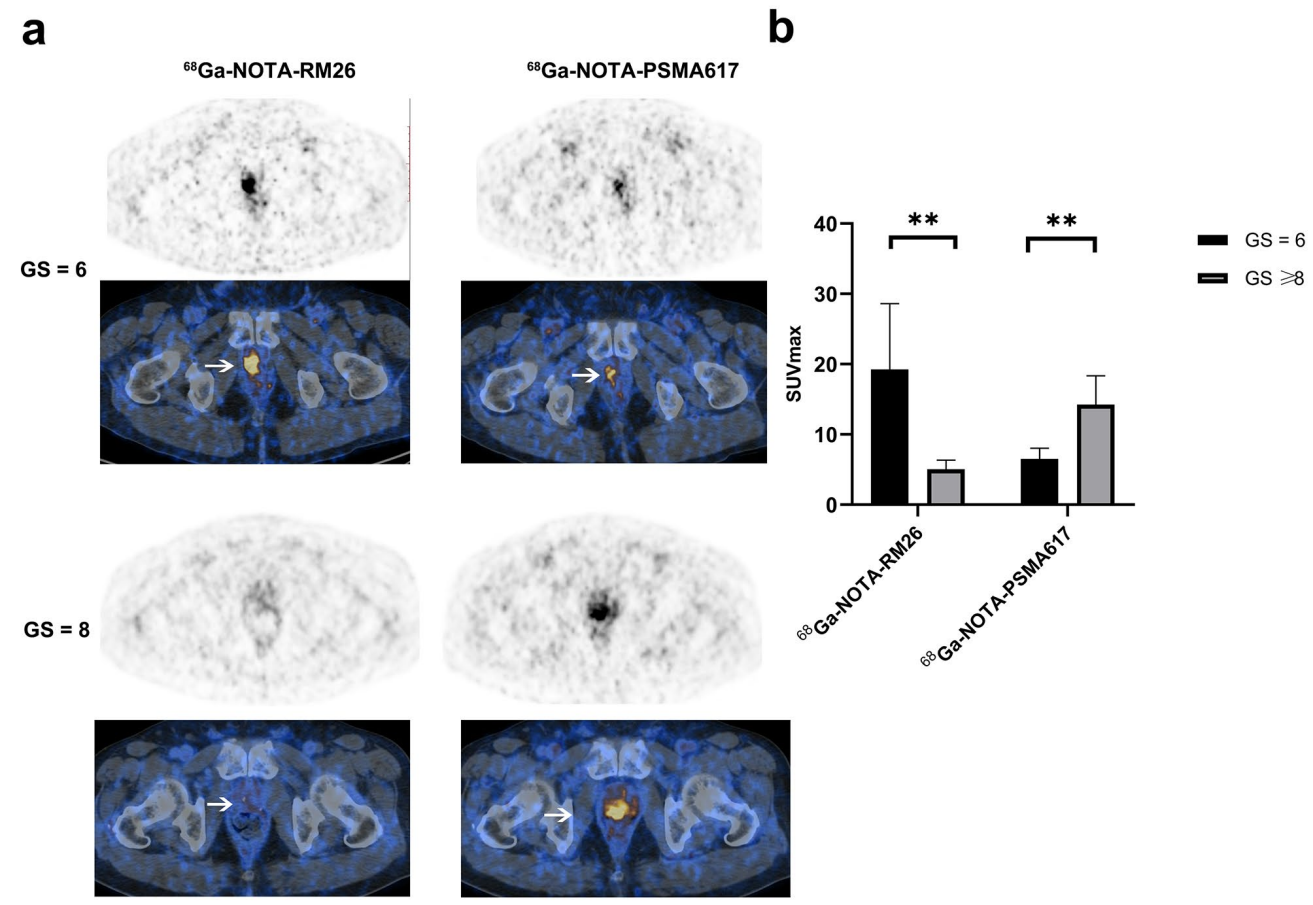
Dual-tracer PET/CT imaging in PCa patients with^68^Ga-NOTA-RM26 and ^68^Ga-NOTA-PSMA617. **a**: Patient 1, a 58-year-old man with PCa (GS = 6). The SUVmax in the primary tumor (white arrow) was 20.1 with ^68^Ga-NOTA-RM26 PET/CT, that was 6.6 with ^68^Ga-NOTA-PSMA617. Patient 2, a 72-year-old man with PCa (GS = 8). The SUVmax in the primary tumor (white arrow) was 3.6 with ^68^Ga-NOTA-RM26 PET/CT and that was 22.3 with ^68^Ga-NOTA-PSMA617. **b**: Quantitative analysis of dual-tracer PET/CT imaging in 16 PCa patients, **, *P* < 0.01

Sixteen patients with histopathologically proven PCa were enrolled (8 with GS = 6, another 8 with GS ≥ 8). As shown in Fig. 7, the PCa patients with high GS (GS ≥ 8) had higher tumor uptake of ^68^Ga-NOTA-PSMA617 (SUVmax:14.26 ± 4.09) than the patients with low GS (GS = 6, SUVmax:6.51 ± 1.53) (*P* = 0.001). Comparatively, the PCa patients with low GS (GS = 6) had higher tumor uptake of ^68^Ga-NOTA-RM26 (SUVmax: 19.26 ± 9.34) than the patients with high GS (GS ≥ 8, SUVmax: 5.06 ± 1.28) (*P* = 0.001).

## Discussion

In this study, we investigated the expression of three biomarkers (GRPR, PSMA, and NTR1) in different stages of precancerous lesions and prostate cancer patients (PIN, PCa, and lymph node metastasis). Our findings revealed that GRPR expression was significantly higher in the stage of PIN and primary prostate cancer with GS = 6, but decreased in lymph node metastasis. PSMA showed high expression in primary lesions and lymph node metastases of prostate cancer, and its level is positively correlated with GS, a poor prognostic indicator. In addition, NTR1 had high expression in primary and metastatic lesions of prostate cancer, but did not show any relationship with clinicopathology and prognosis in patients. PET/CT imaging using these three molecular markers showed promising results and has the potential to be a non-invasive screening methods for prostate cancer.

Early accurate stratified diagnosis of PCa is crucial for clinical management. PSMA-PET/CT imaging is increasingly being employed for the primary staging of PCa due to its superior ability to detect metastatic disease compared with conventional imaging Modalities [21, 22]. The 2024 European Association of Urology (EAU) guidelines recommend the use of PSMA PET/CT for preoperative detection of lymph node metastasis [23]. However, there is no appropriate imaging method for populational screening of early stage of PCa neither for follow-up of patients in active surveillance. Research indicates that PIN, widely considered a premalignant state preceding PCa, may represent early carcinoma in situ with a high likelihood of progressing to invasive PCa [24]. Our study revealed that GRPR expression in PIN was notably higher than PSMA and NTR1. In recent years, studies have shown that the activation of GRP/GRPR pathway plays an important role in the occurrence of prostate cancer [25], suggests that the GRP receptor may be not only a marker, but potentially also a driver of prostatic carcinogenesis, where its over-expression would represent an early event. Radioligand therapy targeting GRPR is expected to play an important role in the early prevention and treatment of prostate cancer. Specifically, ^177^Lu-labeled GRPR antagonists have been developed and show good safety and anti-tumor effects [26, 27]. In primary PCa lesions with GS ≤ 6, GRPR expression was significantly higher than PSMA and progressively increased in PIN and PCa, with a lower expression of only 26.7% in metastatic lesions. These results suggested that GRPR-targeted PET imaging may be suitable for GS = 6 prostate cancer but less suitable for advanced-stage cancer and lymph node metastasis. In this study GRPR expression was high in PC-3 cells, which are not just a metastatic line but also androgen-independent and agressive cancer, However, we believe that this is due to the fact that PC-3 cells are neuroendocrine prostate cancer cells. Although we conducted cell experiments to screen cell lines for establishing xenograft tumor models, further research is needed to verify whether the cell lines can represent the population. Previous studies have reported that PET/CT imaging targeting GRPR has a sensitivity of approximately 70% for lymph node metastasis in prostate cancer patients, but previous study only included three patients with a total of ten lymph node metastases. Therefore, further validation with a larger sample size is necessary to assess the diagnostic value of GRPR PET imaging in lymph node metastasis of prostate cancer [28]. 

The expression of PSMA increased gradually from PIN to primary PCa to lymph node metastasis and was significantly elevated in patients with high GS. This suggests that targeting PSMA imaging might be more suitable for imaging advanced PCa and identifying lymph node metastasis. GRPR was highly expressed in the early stages of PCa and PSMA expression was elevated in advanced PCa, which was consistent with preliminary findings from the PET imaging of the PCa patients. Based on this, we speculated that dual-target imaging of GRPR/PSMA before needle biopsy might improve the detection rate of PCa and enhance the consistency between biopsy pathology and post-radical prostatectomy pathology. Dual-tracer (GRPR + PSMA) PET/CT has shown significant improvements in PCa diagnostic sensitivity, presenting a promising strategy for PCa diagnosis [29]. Previous research found that Dual-tracer PET/CT screen out patients for avoiding 52.67% (59/112) unnecessary biopsy [30]. PCa can be divided according to GS as csPCa (GS = 7–10) and clinically non-significant PCa (cnsPCa, GS = 6). As a result, the recommend treatment strategy for cnsPCa is active surveillance [11], Therefore, noninvasive and accurate detection of prostate cancer patients and Gleason grading prediction are crucial to guide clinical decision-making.

The high expression rate of GRPR in patients with positive surgical margins is significantly higher than that in patients with negative surgical margins. The high expression rate of GRPR in patients with positive surgical margins reaches 95%, and the status of surgical margins is closely related to patient prognosis. However, the high expression rate of PSMA, which represents a late-stage target of prostate cancer, is only 65% in patients with positive surgical margins. We speculate that this may be related to the high proportion of patients with GS ≤ 7 (15/20, 75%) in this group of patients with positive surgical margins, including 3 cases with Gleason score ≤ 6 and 8 cases with GS = 3 + 4. In addition, the number of patients with positive surgical margins in this study is relatively small, and its rationality needs to be verified by larger sample studies. Immunohistochemical analysis revealed a significant correlation between PSMA expression and shorter OS and PFS. Additionally, the intensity of ^68^Ga-PSMA-11 was found to provide prognostic information for adverse pathological outcomes and PFS [31]. There remains uncertainty regarding the relationship between GRPR, PSMA, NTR1 expression, and survival time, suggesting the need for longer follow-up periods to address these limitations.

Although our study found a correlation between high expression of PSMA in advanced PCa and GS, 23 patients with primary lesions with a GS ≥ 7 had no or low expression of PSMA, However, all of these patients have high expression of NTR1, and 15 patients have high expression of GRPR. 2 patients with low expression of PSMA had NED (see Table 2), However, both were positive for GRPR and NTR1, with especially high NTR1 expression that might be related to the lack of PSMA expression in poorly differentiated tumors with NED and downregulation of PSMA expression in neuroendocrine PCa [32]. It has previously been suggested that GRPR and NTR1 may be involved in the progression of NED in PCa [17]. Our preclinical results suggested that GRPR and NTR1 could serve as molecular targets as alternative PET radiopharmaceuticals for the diagnosis and/or therapy in PSMA-negative PCa.

NTR1 has been controversial as a predictor for the survival of cancer patients [33]. Our study found that NTR1 expression in the prostate does not affect PCa patient survival. Also, there was no correlation between NTR1 expression and clinicopathological characteristics of PCa. Moreover, immunohistochemistry analysis showed that despite NTR1 expression progressively increased during PCa development from PIN to PCa, there was no significant difference between primary PCa and lymph node metastasis. Valerie et al. reported that the NTR1 antagonist, SR48692, could inhibit PCa xenograft growth, regardless of whether or not they were androgen-dependent [34]. These findings suggest that targeted NTR1 imaging may serve as an alternative diagnostic imaging method for detecting PSMA-negative prostate cancer.

We explored the expression of GRPR, PSMA, and NTR1 in PCa cell lines and found low expression of PSMA but high expressions of GRPR and NTR1 in PC-3 cells; however, there were increased expressions of GRPR and NTR1 in the DU145 and LNCaP cells. The PC-3 cell line is characteristic of endocrine PCa and expresses high levels of neuroendocrine markers [35], consistent with our observations in two patients with neuroendocrine PCa, who were negative for PSMA and positive for GRPR and NTR1. Wu et al [36] also speculated that high expression of NTR1 is associated with neuroendocrine differentiation of PCa, which makes NTR1 a potential target for neuroendocrine PCa, and PSMA imaging may have false negative in NEPC patients, but targeted NTR1 imaging may make up for the deficiency of PSMA, but their research was limited to cells and animals study. And our findings were mainly based on patient samples. Therefore, we speculate that Therefore, we speculate that the patients with high suspicion of prostate cancer but negative PSMA PET/CT imaging, in addition to considering factors such as ISUP grade and tumour size [37], it should also be considered whether they may be neuroendocrine PCa that need to be verified in follow-up studies.

Additionally, we established a xenograft PCa tumor model and successfully imaged the three agents used. Immunohistochemical staining of mouse tumor tissues confirmed the targeting accuracy of the three probes. Our preliminary study demonstrated that PET/CT imaging may non-invasively stratify PCa patients with different GS levels by targeting different biomarkers.

However, there are several limitations in our study. Firstly, ^68^Ga-NOTA-NT PET imaging in human body has not been carried out, and its imaging effect in human body needs to be further verified. Sencondly, the number of PCa patients included is relatively small, it may have limited somewhat the validity of data analysis. Thirdly, since neuroendocrine markers were only detected in few patients after radical prostatectomy, the effect of neuroendocrine differentiation on the expression of PSMA, GRPR and NTR1 remains to be further verified by studies with more specimens and data.

## Conclusions

On one hand, our study suggested, for the first time, that expression differences of PSMA, GRPR, and NTR1 can reflect different PIN, PCa and lymph node metastasis. PET imaging that targets GRPR is helpful for the diagnosis of early PCa, whereas PSMA is a more appropriate biomarker for high GS disease and metastatic disease and is related to patient prognosis. On the other hand, NTR1 may be suitable for imaging the various stages of PCa. Our study has assessed new molecular probes for PET/CT using three biomarkers (PSMA, GRPR, and NTR1) with potential for non-invasive stratification of PCa patients and potential for translational application in the clinic.

### Electronic supplementary material

Below is the link to the electronic supplementary material.

Supplementary Material 1

## Data Availability

The datasets generated and/or analyzed during the current study are available from the corresponding author upon reasonable request.

## Abbreviations

ADT: Androgen deprivation therapy
GRPR: Gastrin-releasing peptide receptor
GS: Gleason score
NED: Neuroendocrine differentiation
NT: Neurotensin
NTR1: Neurotensin receptor 1
NTRs: Neurotensin receptors
OS: Overall survival
PCa: Prostate cancer
PET: Positron emission tomography
PFS: Progression-free survival
PIN: Prostatic intraepithelial neoplasia
PSA: Prostate-specific antigen
PSMA: Prostate-specific membrane antigen
SUV: Standardized uptake value
tPSA: Total prostate-specific antigen
VOI: Volume of interest
