# Supplementary material for: Evaluation of gastrin-releasing peptide receptor, prostate-specific membrane antigen, and neurotensin receptor 1 as potential biomarkers for accurate prostate cancer stratified diagnosis
Source: EJNMMI Res. 2024 Jun 16;14:55. doi: 10.1186/s13550-024-01116-3 (PMC11180645; doi:10.1186/s13550-024-01116-3)
Supplement: Supplementary file 1 — Supplementary Material 1 [file 13550_2024_1116_MOESM1_ESM.docx]

**Supplementary material:**

Evaluation of gastrin-releasing peptide receptor, prostate-specific membrane antigen, and neurotensin receptor 1 as potential biomarkers for accurate prostate cancer stratified diagnosis

Ling Xiao ^1#^, Zhihui Fang ^1,2#^, Yongxiang Tang ^1^, Yanyan Sun ^3^, Zehua Zhu ^1^, Jian Li ^1^, Ming Zhou ^1^, Nengan Yang ^1^, Kai Zheng ^1^, Shuo Hu ^1,4,5^*

^#^ These authors contributed equally to this work as co-first authors.

**Affiliations**

^1^ Department of Nuclear Medicine, Xiangya Hospital, Central South University, Changsha, 410008, China.

^2^ Department of Nuclear Medicine, The Second Xiangya Hospital, Central South University, Changsha, 410008, China.

^3^ Department of Hematology, The Affiliated Cancer Hospital of Zhengzhou University and Henan Cancer Hospital, Zhengzhou, 450000, China.

^4^ National Clinical Research Center for Geriatric Disorders (Xiangya), Changsha, 410008, China.

^5^ Key Laboratory of Biological Nanotechnology of National Health Commission, Xiangya Hospital, Central South University, Changsha, 410008, China.

*Corresponding author: Shuo Hu, Department of Nuclear Medicine, National Clinical Research Center for Geriatric Disorders, Xiangya Hospital, Central South University, No.87 Xiangya Road, Changsha City, 410008, Hunan Province, P.R. China. E-mail: hushuo2018@163.com

**Reference methods**

**Immunohistochemical staining**

Tissues obtained by transurethral prostate resection, Radical prostatectomy or lymphadenectomy were formalin-fixed and paraffin-embedded before cutting into 3- to 5-µm-thick sections on a microtome. Paraffin sections of the samples were then incubated with anti- GRPR, PSMA, or NTR1 antibodies (ab39883, ab19071, ab117592, Abcam, Cambridge, UK) Rabbit anti-human NTR1 (1:500), mouse anti-human PSMA (1:400), or rabbit anti-human GRPR (1:400) primary antibodies were used as primary antibody for NTR1, PSMA, and GRPR staining.[1] Staining without the primary antibody was also performed to confirm its specificity as a negative control. The goat anti-rabbit immunoglobin G (IgG) secondary antibody (1:200) was used and incubated at room temperature (20–25 °C) for 50 minutes for the NTR1 group. For the PSMA and GRPR groups, mouse- and rabbit-specific HRP/DAB detection kits were used, respectively. The expression of GRPR, PSMA, and NTR1 proteins were assessed using a 4-tiered intensity score system (negative, weak, moderate, and strong). Two independent pathologists blinded to the clinicopathologic information performed the scorings. Negative and weak groups were categorized as low-expression groups, while moderate and strong groups were categorized as high-expression groups.

**RT-PCR**

RT-PCR analysis was performed using the TB Green Premix Ex Taq Ⅱ (TaKaRa Bio Inc, Shiga, Japan) on a LightCycler 96 system (Roche, Basel, Switzerland). The relative fold changes of candidate genes were analyzed using the 2^−ΔΔCt^ method. GRPR expression was assessed using the forward primer 5’-CGGAAGCGACTTGCCAAGACAG-3’ and the reverse primer 5’-GAGTAGTGGTAGGAGCGGTACAGG-3’, PSMA expression was assessed using the Forwardprimer 5’-GTTAATTGCAGAGGCTGTTGGTC-3’, and the reverse primer 5’-GCTGCTATCTGGTGGTGCTGAG-3’, and NTR1 expression was assessed using the forward primer 5’-CAAGACCGTGAAGAGTTGAAGGC-3’ and the reverse primer 5’-TTGTGCTTGTGGCTGATCTGACTG -3’ (Sangon Biotech, Shanghai, China).

**Western blotting**

Western blotting was used to evaluate the expression of GRPR, PSMA, and NTR1 in PC-3, LNCaP and DU145 cells. Samples (20μg per lane) were separated by 12% sodium dodecyl sulfate polyacrylamide gel electrophoresis (SDS-PAGE) and transferred to polyvinylidene fluoride (PVDF) membranes. Membranes were blocked with 5% defatted milk for 1.5 h at room temperature (temperature of 20-25°C) and, then incubated overnight at 4°C with primary antibodies: rabbit anti-NTR1 (1:1000 dilution, Abcam, ab117592), rabbit anti-GRPR (1:1000 dilution, Abcam, ab39883), mouse anti-PSMA (1:1000 dilution, Abcam, ab19071) and rabbit anti-GAPDH (1:2000 dilution, Proteintech). The relative expression levels of protein were normalized by the ratio of target protein (PSMA, GRPR and NTR1) to GAPDH.

**Radiolabeling and quality control**

The precursor GRPR(NOTA-RM26) and NTR1(NOTA-NT) were obtained from GL Biochem Company, the precursor PSMA(NOTA-PSMA617) was obtained from Huayi Isotopes Company. GRPR, PSMA and NTR1 were radiolabeled with an automated module (ITM). ^68^Ga-NOTA-RM26, ^68^Ga-NOTA-PSMA617, and ^68^Ga-NOTA-NT was prepared according to a previously published method[2]. Thirty µg precursor compounds (NOTA-RM26, NOTA-PSMA617, and NOTA-NT) were mixed with 1 ml of 0.25 mol/L NaOAc aqueous solution, 1110–1295 MBq of ^68^Ga was added to the reaction tube with 4 ml HCl (0.05 mol/L), and incubated at 90℃ for 10 min. Subsequently, 10 ml of deionized water was added to quench the reaction, and the product was purified by passing the reaction mixture over a Sep-Pack C18 cartridge. Next, 1 ml of ethanol and 10 ml of normal saline were used successively to wash and filter the product injection. The purity of labeled products was analyzed by HPLC using the chromatographic column ZORBAX SB-C18: mobile phase A was distilled water containing 0.1% TFA and mobile phase B was acetonitrile containing 0.1% TFA. Gradient elution was performed with 5% acetonitrile for 0-2min and 90% acetonitrile for 3-15 min. The peak times of ^68^Ga NOTA-RM26, ^68^Ga-NOTA-PSMA617, and ^68^Ga-NOTA-NT were 8.72, 7.32, and 10.89 min, respectively (**Supplemental Figure 1, Supplemental Figure 2**). The purity of the products was more than 99%.

**Animal imaging**

PET imaging analysis was performed when the tumor size reached approximately 500–900 mm3 (LNCaP mice xenograft around 6–8 weeks after cell inoculation, PC-3 mice xenograft around 4–6 weeks after cell inoculation). Mice were anesthetized by isoflurane inhalation before injecting 3.7 MBq of ^68^Ga-NOTA-RM26, ^68^Ga-NOTA-PSMA617, or ^68^Ga-NOTA-NT. Small-animal PET/CT (Mediso, Budapest, Hungary) imaging was performed 30 minutes after the injection. For visualization of ^68^Ga-NOTA-RM26, ^68^Ga-NOTA-PSMA617, and ^68^Ga-NOTA-NT uptake in different organs, PET images were decay-corrected using the half-life of ^68^Ga(68mins) and normalized using the standardized uptake (SUV) factor defined as injected dose (kBq) per g body weight. To calculate ^68^Ga-NOTA-RM26, ^68^Ga-NOTA-PSMA617, and ^68^Ga-NOTA-NT SUV uptake in the tumor, regions of interest were drawn to define the volume of interest (VOI, mL) of the tumor in each mouse. The xenograft was soaked in 4% paraformaldehyde after imaging. After fixation, dehydration, paraffin embedding, and sectioning, the expression of GRPR, PSMA, and NTR1 was detected by immunohistochemical staining using the following primary antibodies: rabbit anti-GRPR (ab39883, Abcam), rabbit anti-PSMA (13163-1-AP, Proteinch, USA), mouse anti-NTR1 (sc-393205, Santa Cruz Biotechnology, USA).

Supplemental Figure 1 Chemical structure of ^68^Ga-NOTA-RM26, ^68^Ga-NOTA-PSMA617, and ^68^Ga-NOTA-NT


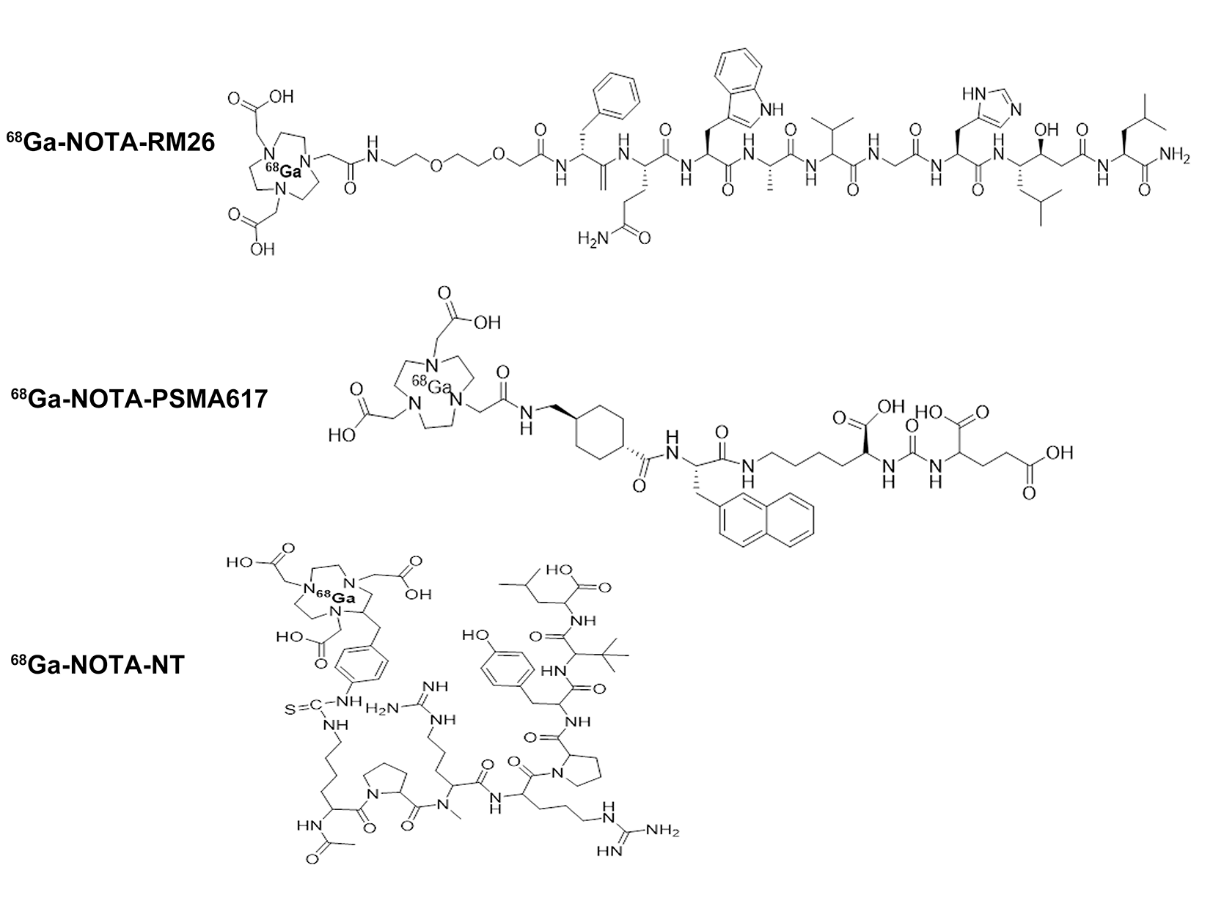


Supplemental Figure 2 HPLC analysis of ^68^Ga-NOTA-RM26, ^68^Ga-NOTA-PSMA617, and ^68^Ga-NOTA-NT

**
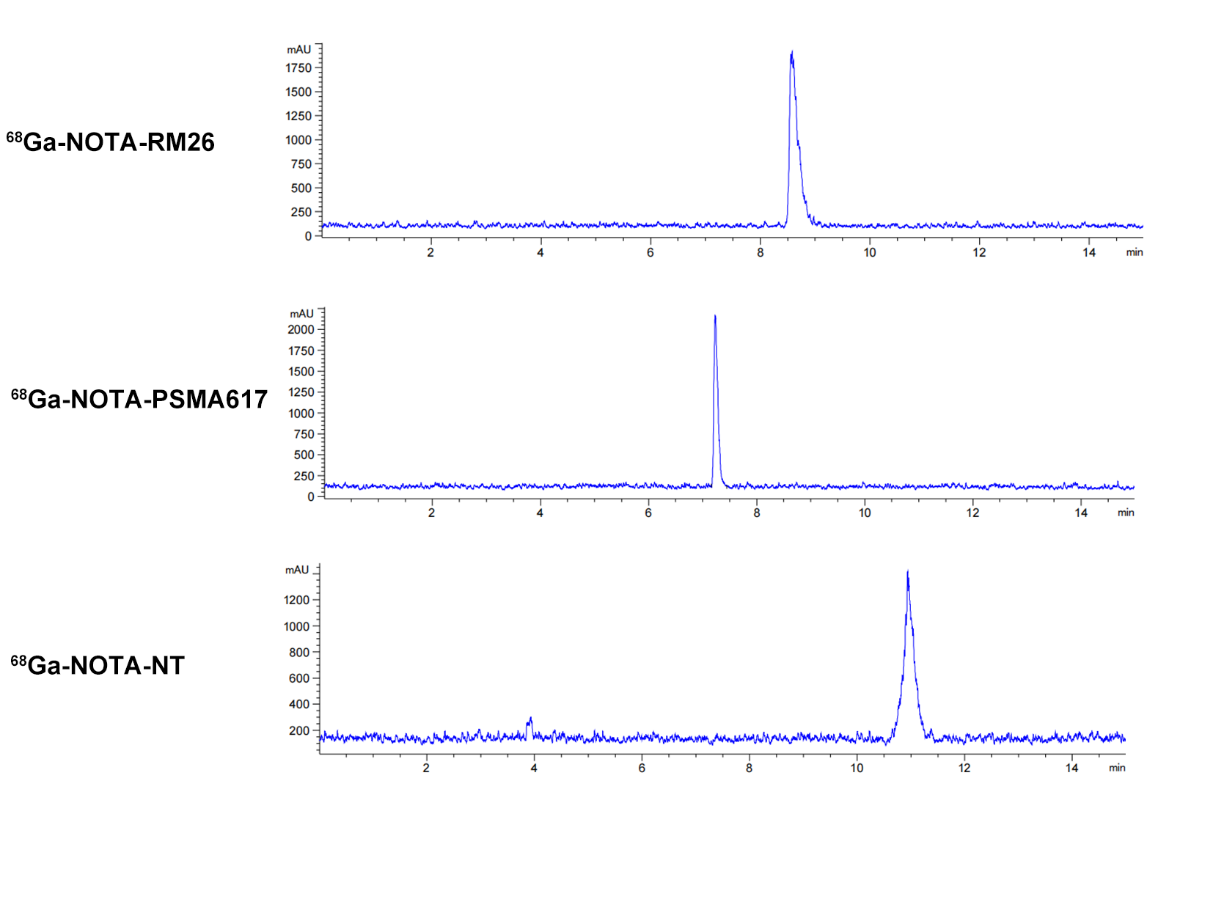
**

Supplemental Figure 3 The uncropped western blots. We cropped the picture height and width to make the image more beautiful, and did not edit the data arrangement. the uncropped gels belonging to figure 5-c panel.
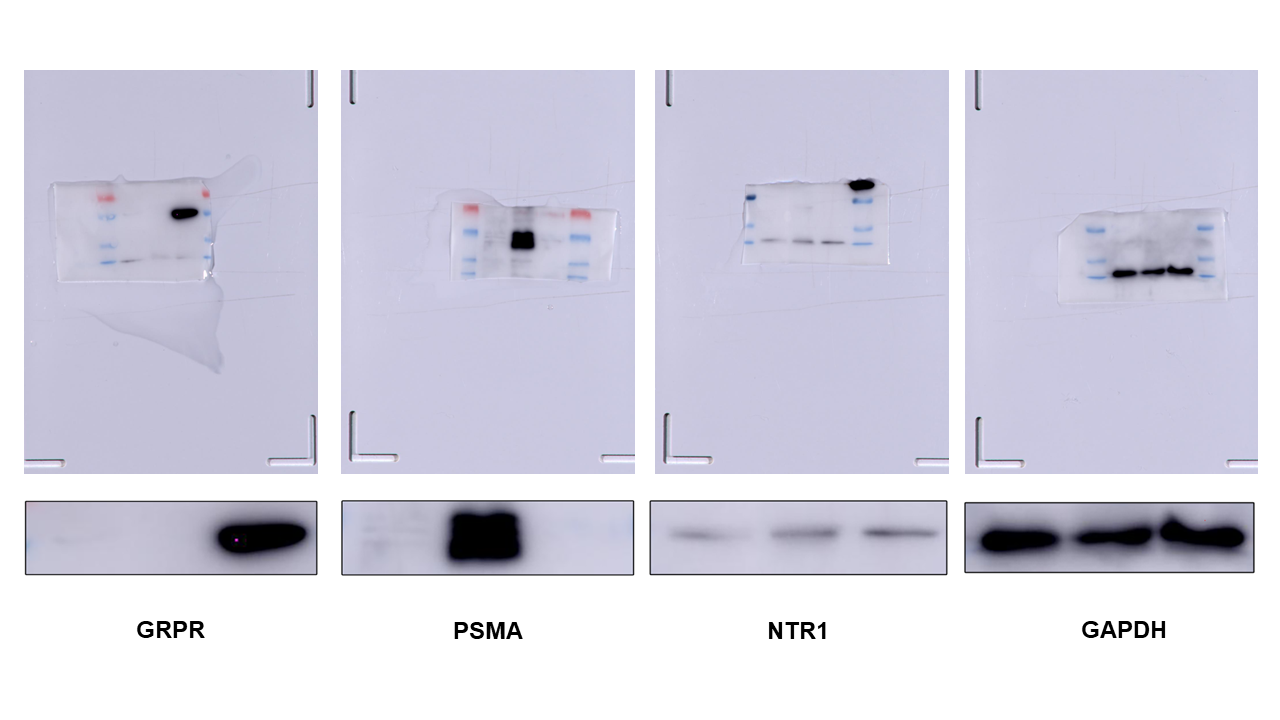


1. Yin X, Wang M, Wang H, Deng H, He T, Tan Y, et al. Evaluation of neurotensin receptor 1 as a potential imaging target in pancreatic ductal adenocarcinoma. Amino Acids. 2017;49:1325-35. doi:10.1007/s00726-017-2430-5.

2. Greifenstein L, Engelbogen N, Lahnif H, Sinnes JP, Bergmann R, Bachmann M, et al. Synthesis, Labeling and Preclinical Evaluation of a Squaric Acid Containing PSMA Inhibitor Labeled with (68) Ga: A Comparison with PSMA-11 and PSMA-617. ChemMedChem. 2020;15:695-704. doi:10.1002/cmdc.201900559.
